# Ellagic Acid: A Dietary-Derived Phenolic Compound for Drug Discovery in Mild Cognitive Impairment

**DOI:** 10.3389/fnagi.2022.925855

**Published:** 2022-07-04

**Authors:** Wenjun Wang, Shaohui Wang, Yue Liu, Xiaobo Wang, Jia Nie, Xianli Meng, Yi Zhang

**Affiliations:** ^1^State Key Laboratory of Southwestern Chinese Medicine Resources, School of Ethnic Medicine, Chengdu University of Traditional Chinese Medicine, Chengdu, China; ^2^State Key Laboratory of Southwestern Chinese Medicine Resources, School of Pharmacy, Chengdu University of Traditional Chinese Medicine, Chengdu, China

**Keywords:** ellagic acid (EA), mild cognitive impairment (MCI), sources, physicochemical properties, pharmacokinetics, mechanism

## Abstract

Ellagic acid (EA), a naturally occurring polyphenolic compound, is detected in free form or linked to polyols or sugars, constituting hydrolyzable tannins or ellagitannins in distinct fruits, nuts, and herbs. Today, a considerable number of botanicals and enriched foods containing EA are commercially available as nutraceuticals and used to prevent mild cognitive impairment (MCI) due to the excellent neuroprotective capacity of EA. Here, this study aims to provide an overview of the physicochemical properties, source, and pharmacokinetics of EA and to emphasize the importance and mechanisms of EA in the prevention and management of MCI. To date, preclinical studies of EA and its derivatives in various cell lines and animal models have advanced the idea of dietary EA as a feasible agent capable of specifically targeting and improving MCI. The molecular mechanisms of EA and its derivatives to prevent or reduce MCI are mainly through reducing neuroinflammation, oxidative stress, neuronal apoptosis, synaptic dysfunction and loss, and defective mitochondrial functions. Nevertheless, well-designed and correctly large randomized controlled trials in the human population need to be performed to reinforce the scientific facticity of the beneficial effects of EA against MCI. Synchronously, the mechanism of EA against MCI is least provided cynosure and expects more attention from the emerging research community.

## Graphical Abstract

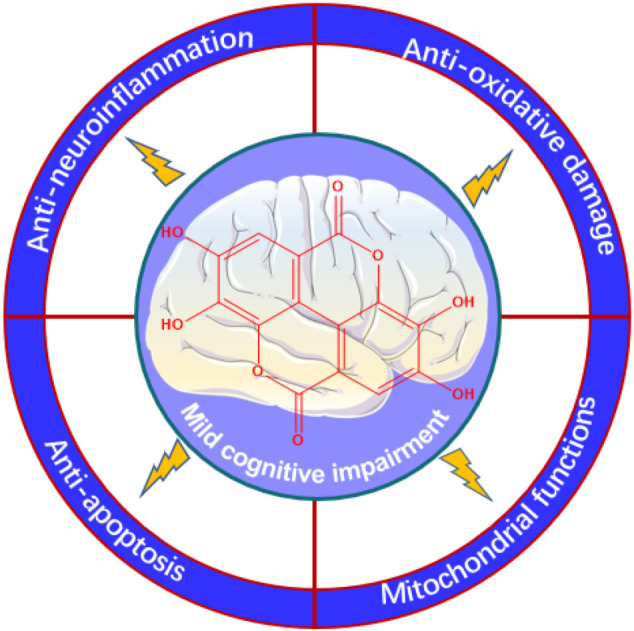

## Introduction

The global rise in life expectancy is resulting in a dramatic increase in the size of the elderly population, with the number of people aged 60 years and over expected to rise from 600 million in 2000 to 2.1 billion globally by 2050 and this will have a major impact on the health, social, and economic sectors (Ellen et al., 2017). Mild cognitive impairment (MCI) is usually associated with cognitive impairment, which is seen with general age-related cognitive decline, but is not severe enough to lead to considerably impaired daily function (Stevens et al., 2022). Furthermore, the principal cognitive domain that could be afflicted refers to a decline in the capability to acquire raw information or recall stored information (Gatz et al., 2022; Kuroda et al., 2022). Clinically, age-related cognitive impairment represents the preclinical, transitional stage between healthy aging and dementia (Levey et al., 2021). In addition, other factors such as sleep deprivation, depression, or diabetes mellitus can also induce cognitive impairment (Guan et al., 2020; Yu et al., 2021). Since Petersen et al. (1997) first comprehensively described the characteristics of MCI in 1997, scholarly and clinical interests in MCI have grown exponentially. More than 55 million people all over the world are suffering from dementia (WHO, 2021). It is expected that the worldwide prevalence of dementia will nearly double every 20 years, with the number of patients expected to rise to 131 million by 2050 if breakthroughs are not made to prevent the disease or delay its onset (Martins et al., 2018). However, in the last 10 years, drug trials have failed 99.6% of the time because existent drug therapies for neurodegenerative diseases rarely curtail the underlying disease processes (Anderson, 2019).

Given this, researchers have shifted focus toward the early steps to counteract or, anyway, retard the progression from MCI to dementia, which would profoundly reduce the prevalence and costs of neurodegenerative diseases (Jongsiriyanyong and Limpawattana, 2018; Zhang et al., 2019). Therefore, it is mandatory to find more efficacious preventive strategies to straightly prevent, decelerate, and even discontinue this “dementia pandemic.” Physical exercise and healthy dietary habits have been identified as some of the most efficient therapeutic approaches and are recommended from early life (Blumenthal et al., 2017). In addition, the connection between diet, health, and the presence of bioactive chemical compounds in food has gotten fantastic attention in recent years and consumers are progressively interested in food products that, apart from fulfilling nutritional demands, enhance physical performance, further wellbeing, and reduce the risk of producing diseases (Auxtero et al., 2021; Ozawa et al., 2021). Nutritional interventions or botanicals such as polyphenols can be a greatly accepted, inexpensive, and efficacious option to preserve against age-related cognitive decline and neurodegeneration, leading to important personal and societal benefits (Tewari et al., 2018; de Araujo et al., 2021). Synchronously, considering the transformation at the colonic level and absorption, the good bioavailability of some polyphenols owing to the ability to pass the blood–brain barrier, to a various extent, facilitated the interest in these molecules as fresh neuroprotective means against neurodegenerative disorders (Yan et al., 2022; Yuste et al., 2022).

Ellagic acid (EA), a polyphenol presents in fruits, nuts, and herbs, owns some salient pharmacological properties (Usta et al., 2013; Gupta et al., 2021). EA is also commercially available as nutraceuticals and used to protect against chronic diseases such as diabetes (Altindag et al., 2021) and cardiovascular (Mansouri et al., 2020) and neurodegenerative diseases (Kumar et al., 2021). In this circumstance, EA invited researchers’ interest for its expected health benefits in pathological statuses of MCI and, currently, several literature data give support to such EA capability (Alfei et al., 2019).

This narrative review aims to summarize the physicochemical properties, source, and pharmacokinetics of EA and the most relevant evidence of the effects of EA on MCI. The potential mechanisms of action underlying the neuroprotective activity of EA, provided from animal or *ex-vivo* research, are discussed to consider knowing the therapeutic potential of EA and are expected to lay the solid foundation for the clinical application of EA in the prevention and management of MCI.

## Physicochemical Properties and Sources of Ellagic Acid

Ellagic acid, likewise recognized as a dimeric derivative of gallic acid, is a dietary-derived phenolic compound with a chemical formula of C_14_H_6_O_8_ and from the physicochemical perspective, in the form of cream-colored needles or yellow powder with a melting point greater than 360°C, a boiling point of 796.50°C, a log P of 1.05, and a molecular weight of 302.194 g/mol (Garcia-Nino and Zazueta, 2015; Shakeri et al., 2018). EA is identified as 6,7,13,14-tetrahydroxy-2,9 dioxatetracyclo[6.6.2.0^4,16^.0^11,15^]hexadeca-1(15),4,6,8(16),11,13-hexaene-3,10-dione according to IUPAC nomenclature. The chemical structure of EA is composed of four free ⋅OH groups and two acyloxy groups standing for the hydrophilic moiety and a lipophilic planar moiety comprising two hydrocarbon phenyl rings (Sharifi-Rad et al., 2022). The structure of EA influences the solubility and, hence, the bioavailability. Indeed, EA is easily soluble in methanol (671 μg/ml, temperature at 37°C), polyethylene glycol 400, alkalies, dimethyl sulfoxide, triethanolamine, or pyridine, slightly soluble in alcohol (200 μg/ml, temperature at 37°C) or water (about 9.3–9.7 μg/ml, temperature at 21°C, and pH 7.4), practically insoluble in ether, since from both its planar and symmetrical structure and a broad network of hydrogen bonds (Evtyugin et al., 2020). The detailed physicochemical properties of EA are shown in Table 1.

**TABLE 1:** Chemical and physical properties of ellagic acid (EA).

Chemical name	Ellagic acid
	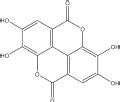
**2D structure**	
CAS number	476-66-4
Canonical SMILES	C1 = C2C3 = C(C( = C1O)O)OC( = O)C4 = CC( = C(C( = C43)OC2 = O)O)O
IUPAC name	6,7,13,14-tetrahydroxy-2,9 dioxatetracyclo[6.6.2.0 ^4,16^.0^11,15^]hexadeca-1(15),4,6,8(16),11,13-hexaene-3,10-dione
Molecular weight	302.194 g/mol
Molecular formula	C_14_H_6_O_8_
Color/Form	Cream-colored needles or yellow powder
Melting point	Greater than 360°C
Solubility	Slightly soluble in alcohol or water, soluble in alkalies, in pyridine. Practically insoluble in ether
Density	1.667 at 64 °F
Hydrogen bond donor count	4
Hydrogen bond acceptor count	8
Covalently bonded unit count	1
Dissociation constants	pKa_1_ = 6.69 (phenol); pKa_2_ = 7.45 (phenol); pKa_3_ = 9.61 (phenol); pKa_4_ = 11.50 (phenol)
UV spectra	UV_max_ (ethanol): 366, 255 nm
ADMET BBB level	4
Druglikeness weight	0.327

**FIGURE 1:**
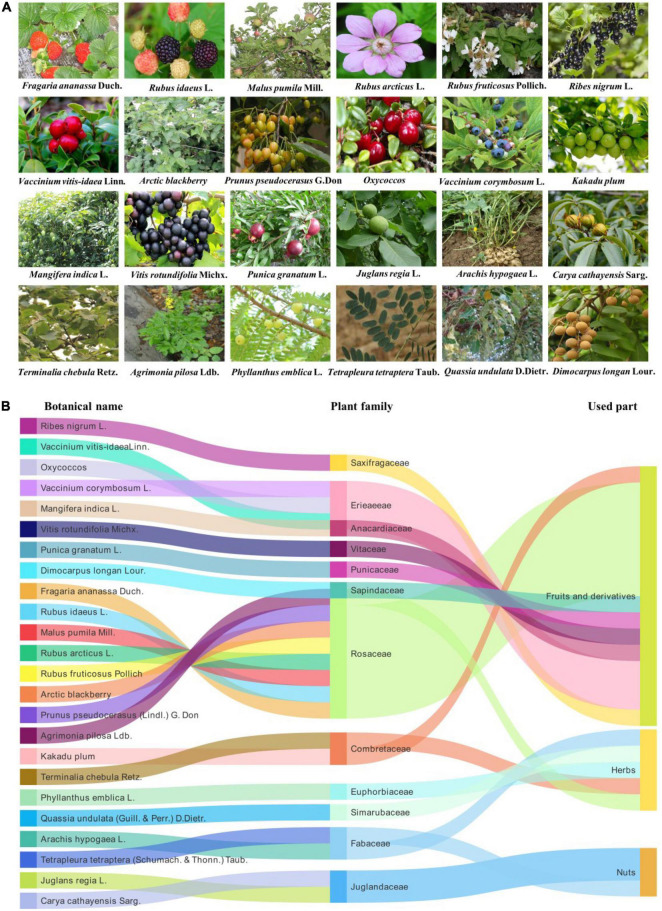
Various sources of ellagic acid (EA). **(A)** The plant origins of EA. The pictures are from the website: https://www.gbif.org/; http://www.iplant.cn/frps. **(B)** EA as a major constituent of different fruits, nuts, and herbs.

Our investigation shows that, so far, EA is isolated from the family Rosaceae, Saxifragaceae, Ericaceae, Combretaceae, Anacardiaceae, Vitaceae, Punicaceae, Juglandaceae, Fabaceae, Euphorbiaceae, Sapindaceae, and Simarubaceae. Especially, EA is primarily plentiful in berries of the family Rosaceae such as raspberry, cherry, and strawberry. Herein, Figure 1 displays a comprehensive list of EA sources, arranged in fruits and derivatives, nuts, and herbs. Among these, this plant-based provision can embody EA in three distinct forms: free EA, ellagic acid glycosides, and polymeric ellagitannins even if in varying quantities (Lee et al., 2005). In fact, EA is predominately detected as ester-linked to sugars in the composition of hydrolyzable tannins that are called ellagitannins (Long et al., 2019). For instance, a strawberry yield 70 mg of ellagitannins, approximately 100 g of raspberry produces 300 mg of ellagitannins, and four walnuts produce 400 mg of ellagitannins (Aishwarya et al., 2021). Nevertheless, free EA is present in significant amounts in kakadu plums, raspberry fruit, and pomegranate. Among them, the content of EA in the kakadu plum is the highest, ranging from 8.26 to 14.7 mg/g of fresh fruit weight (Table 2).

**TABLE 2:** Sources of ellagic acid (EA) and its content in different fruits, nuts, and herbs.

Plant family	Plant species	Botanical name	Used part	Amount isolated	References
Anacardiaceae	Mango	*Mangifera indica* L.	Fruits and derivatives	1.20 mg/g	Soong and Barlow, 2006
Combretaceae	Kakadu plum	*Kakadu plum*	Fruits and derivatives	Whole fruit: 8.26–14.7 mg/g; Puree: 6.15–13.31 mg/g	Williams et al., 2016
	Terminalia chebula	*Terminalia chebula* Retz.	Herbs	8.00 mg/g	Pellati et al., 2013
Erieaeeae	Bilberry	*Vaccinium vitis-idaea* Linn.	Fruits and derivatives		FooDB, 2020
	Cranberry	*Oxycoccos*	Fruits and derivatives	0.12 mg/g	Daniel et al., 1989
	Highbush blueberry	*Vaccinium corymbosum* L.	Fruits and derivatives	0.0014 mg/g	FooDB, 2020
Euphorbiaceae	Phyllanthus amarus	*Phyllanthus emblica* L.	Herbs		Alagan et al., 2019
Fabaceae	Tetrapleura tetraptera	*Tetrapleura tetraptera* Taub.	Herbs		Odubanjo et al., 2018
	Peanut	*Arachis hypogaea* L.	Nuts		FooDB, 2020
Juglandaceae	Walnut	*Juglans regia* L.	Nuts	0.59 mg/g	Daniel et al., 1989
	Pecans	*Carya cathayensis* Sarg.	Nuts	0.33 mg/g	Daniel et al., 1989
Punicaceae	Pomegranate	*Punica granatum* L.	Fruits and derivatives	8.61 mg/g External peels: 28.53 mg/g Juice: 0.0206 mg/mL	Masci et al., 2016
Rosaceae	Strawberry	*Fragaria ananassa* Duch.	Fruits and derivatives	0.683–0.853 mg/g	Koponen et al., 2007
	Raspberry	*Rubus idaeus* L.	Fruits and derivatives	2.637–3.309 mg/g Juice: 0.0084 mg/mL	Koponen et al., 2007; Neveu et al., 2010
	Apple	*Malus pumila* Mill.	Fruits and derivatives		FooDB, 2020
	Arctic bramble	*Rubus arcticus* L.	Fruits and derivatives	3.9 mg/g	Maatta-Riihinen et al., 2004
	Blackberry	*Rubus fruticosus* Pollich	Fruits and derivatives	1.5 mg/g	Daniel et al., 1989
	Arctic blackberry	*Arctic blackberry*	Fruits and derivatives	0.1715 mg/g	Neveu et al., 2010
	Cherry	*Prunus pseudocerasus* G. Don	Fruits and derivatives		FooDB, 2020
	Agrimonia pilosa	*Agrimonia pilosa* Ldb.	Herbs		Kashchenko and Olennikov, 2020
Sapindaceae	Dimocarpus longan	*Dimocarpus longan* Lour.	Fruits and derivatives		FooDB, 2020
Saxifragaceae	Blackcurrant	*Ribes nigrum* L.	Fruits and derivatives		FooDB, 2020
Simarubaceae	Quassia undulata	*Quassia undulata* D.Dietr.	Herbs		Odubanjo et al., 2018
Vitaceae	Muscadine grape	*Vitis rotundifolia* Michx.	Fruits and derivatives	Whole fruit: 0.0092 mg/g; Juice: 0.009–0.0093 mg/mL	Neveu et al., 2010; FooDB, 2020

## Pharmacokinetics of Ellagic Acid

**FIGURE 2:**
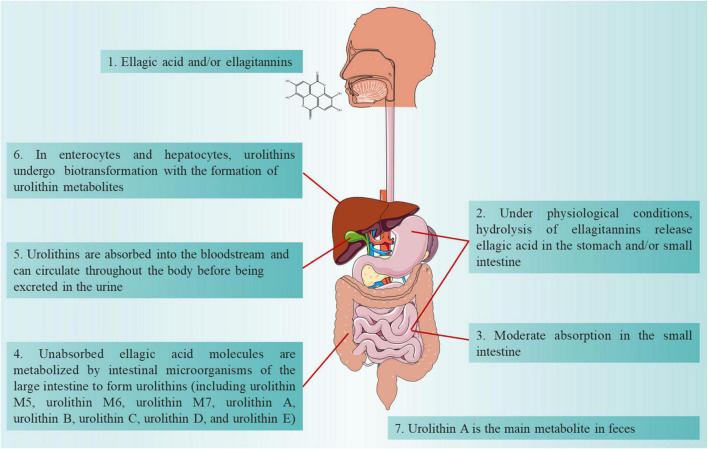
Absorption, distribution, metabolism and excretion processes of ellagic acid (EA) in the body.

An in-depth understanding of the pharmacokinetic properties of EA is helpful to understand its absorption, distribution, metabolism, and excretion processes in the body. Ellagitannins-rich foods are metabolically hydrolyzed in the gastrointestinal tract, releasing the amount of free EA, which is then absorbed in the body or undergoes a massive metabolism to yield a series of urolithins (dibenzopyran-6-one metabolites) by the gut microbiota, including urolithin M5, urolithin M6, urolithin M7, urolithin A, urolithin B, urolithin C, urolithin D, and urolithin E (Figure 2; Garcia-Villalba et al., 2013; Garcia-Nino and Zazueta, 2015; Watanabe et al., 2020). Among EA-derived metabolites, urolithin A is the major metabolite that is discovered in fecal matter in human beings (Gonzalez-Barrio et al., 2011; Jayatunga et al., 2021). The amount of EA in the peripheral tissues and systemic circulation is virtually paltry, while urolithins and their conjugates can extend to concentrations at the micromolar point (Cerda et al., 2005). Plasma levels of EA after the intake of vegetables and fruits (normal plasma concentrations of 0.1–0.4 μmol/l) are restricted by poor water solubility and bioavailability of EA (Seeram et al., 2004; Murugan et al., 2009). However, a growing number of studies are incessantly concentrated on arranging water-soluble and absorbable EA formulations, including drug delivery systems (i.e., nanoparticles, liposomes, microemulsions, and polymeric) and novel formulations (i.e., particle size reduction, amorphization, and lipid-based formulations), to ameliorate significantly the bioavailability and solubility of EA (Mady and Ibrahim, 2018; Nyamba et al., 2021). Furthermore, despite the therapeutic capability of available delivery systems to improve the bioavailability of EA, new and advanced formulations that can eliminate the hepatic first-pass pharmacokinetic impact and uphold superlative efficacious concentrations of EA in the systemic circulation are worthy.

## Therapeutic Properties of Ellagic Acid in Mild Cognitive Impairment

Although a large number of environmental and genetic factors are associated with MCI, the pathological mechanism continues to be unclear. In recent years, numerous animal model studies have been used to examine the potential pharmacological effects of EA on MCI (Table 3). Thus, the evidence for the behavioral consequences of EA in animal models has improved considerably.

**TABLE 3:** Animal behavioral tests representing the effects of ellagic acid (EA) against mild cognitive impairment (MCI).

Sl./No.	Animal model	EA dosage and treatment duration	Behavioral tests	References
I	Streptozotocin-induced MCI			
1	Wistar rats	EA (50 mg/kg, p.o.) for 30 days	Y-maze, Radial arm maze	Jha et al., 2018
2	Wistar rats	EA (35 mg/kg, p.o.) for 4 weeks	Morris water maze test, Elevated plus maze test	Kumar and Bansal, 2018
II	Lipopolysaccharides-induced MCI			
1	Wistar rats	EA (100 mg/kg, p.o.) for 8 days	Open field test, Novel object recognition test	Dornelles et al., 2020
2	Wistar rats	EA 218.06 μg/mL: quantitative analysis of *Phyllanthus amarus* (200 and 400 mg/kg, p.o.) for 14 and 28 days	Novel object recognition test	Alagan et al., 2019
3	C57BL/6 mice	Ellagitannin: geraniin (20 mg/kg, p.o.) for 14 days	Morris water maze test	Wang et al., 2019
III	Transgenic animals-induced MCI			
1	R6/2 mice	EA (50 and 100 mg/kg, p.o.) for 4 weeks	Novel object recognition test, Y-maze test, Open field test	Sun et al., 2020
2	APP/PS1 transgenic mice	EA (50 mg/kg, p.o.) for 60 days	Morris water maze test	Zhong et al., 2018
3	ICR mice	EA from persimmon (50 and 100 mg/kg, p.o.) for 3 weeks	Y-maze test, Passive avoidance test, Morris water maze test	Yoo et al., 2021
4	ICR mice	Urolithin A-the metabolite of EA (50, 100, and 150 mg/kg, p.o.) for 8 weeks	Morris water maze test, Novel object recognition test	Chen et al., 2019
5	APP/PS1 mice	Urolithin A-the metabolite of EA (300 mg/kg, p.o.) for 14 days	Morris water maze test	Gong et al., 2019
IV	Scopolamine- and diazepam-induced MCI			
1	Wistar rats	EA (30 and 100 mg/kg, i.p.) for 10 days	Elevated plus-maze test, Passive avoidance test, Open field test	Mansouri et al., 2016
2	Wistar rats	EA from *Tetrapleura tetraptera* and *Quassia undulata* (50 and 300 mg/kg, p.o.) for 14 days	Novel object recognition test, Y-maze test	Odubanjo et al., 2018
V	Other facts-induced MCI			
1	traumatic brain injury (TBI) in rat	EA (100 mg/kg, i.p.) for 3 days	Passive avoidance test	Mashhadizadeh et al., 2017
2	Symptoms of HD rats (3-nitropropionic acid)	EA (25, 50, and 100 mg/kg, p.o.) for 21 days	Novel object recognition test, Elevated plus maze test	Sharma et al., 2021

### Behavioral Effects of Ellagic Acid in Models of Streptozotocin-Induced Mild Cognitive Impairment

Streptozotocin (STZ) is glucosamine-nitrosourea, which demonstrates insulin resistance-related brain abnormalities when administered intracerebroventricular (ICV) at a subdiabetogenic dose. These abnormalities include cognitive impairment, impaired glucose and energy metabolism in the brain, amyloid β (Aβ) deposition, neuroinflammation, and other neurochemical and neuropathological changes that ultimately lead to memory and learning disabilities similar to those present in sporadic Alzheimer’s disease (AD). Therefore, ICV-STZ can be used to establish experimental animal models of early pathological changes in sporadic AD (Noor et al., 2022). The insulin signaling cascade in the hippocampus is extremely significant for cellular metabolism and neuron survival and is associated with cognitive functions (Zeng et al., 2016). Furthermore, these involve defective mitochondrial functionalities, oxidative stress injury, and consequential cytoenergetic catastrophe, which are correlated with declined cognitive role through a modification in the architecture of the neural network. Administration with EA (50 mg/kg, po) for 30 days can distinctly ameliorate MCI caused by streptozotocin (Jha et al., 2018). Meanwhile, administration of EA (35 mg/kg, po) for 4 weeks notably attenuated the neurotoxic influence of streptozotocin in the rat brain and thereby revealed memory-enhancing functions (Kumar and Bansal, 2018).

### Behavioral Effects of Ellagic Acid in Models of Lipopolysaccharides-Induced Mild Cognitive Impairment

Neuroinflammation is a predisposing factor for the development of MCI (Merlini et al., 2019). Abnormal neuroimmune signaling is associated with cognitive dysfunction and systemic lipopolysaccharides (LPSs) administration has been identified as experimental model that simulates the pathological disorders of MCI. LPS can damage the consolidation of exact memory processes. Intraperitoneal (IP) injection of LPS causes cognitive impairment in experimental animals by activating microglia, which stimulates the production of proinflammatory mediators (Dornelles et al., 2020; Woodcock et al., 2021). On one hand, acute administration of LPS before practice harms the test of situational fear conditioning, a hippocampal-dependent learning paradigm, whereas chronic LPSs injection influences spatial memory and leads to impairment of memory and learning. On the other hand, systemic administration of LPS led to impairing the hippocampal-dependent object recognition memory, but did not affect spatial memory. Study employing this model has expected that IP application of LPS is able of inducing MCI in laboratory animals, while treatment with 100 mg/kg of EA for 8 days was capable of significantly preventing MCI, which was assessed through the open field and object recognition tests (Dornelles et al., 2020). In fact, geraniin, as a major ellagitannin, has the same effect on MCI as EA. This study showed that daily intragastrical administration with geraniin (20 mg/kg) for 14 days significantly mitigated LPS-elicited cognitive impairment in mice (Wang et al., 2019). Meanwhile, EA from *Phyllanthus amarus* Schum. and Thonn. that was given the dose of 200 and 400 mg/kg (for 14 and 28 days) effectively protected the rodents from LPS-induced MCI (Alagan et al., 2019).

### Behavioral Effects of Ellagic Acid in Models of Transgenic Animals-Induced Mild Cognitive Impairment

A large number of studies disclosed that EA exerted neuroprotective properties on several neurodegenerative dysfunctions, including AD, Huntington’s disease (HD), and Parkinson’s disease (PD). R6/2 mice simulated human HD by expressing part of the human HD gene [1 Kb 5′ untranslated region (UTR) sequence and 1 exon, approximately 120 CAG repeats] under the promoter element of the human gene. In short, a progressive neurological phenotype mimicking some features of HD in humans with early symptoms such as verbal fluency, mild word-finding difficulty, and cognitive impairment onset at 15–21 weeks is revealed by R6/2 mouse line. Sun et al. (2020) reported that both the high dose and low dose of EA (50 and 100 mg/kg, single oral gavage for 4 weeks) significantly attenuated MCI and ameliorated neuropathological features in R6/2 mice. Meanwhile, 3-nitropropionic acid is a natural systemic toxin, which is used to cause MCI in experimental animals with the HD model. Nevertheless, administration of EA (25, 50, and 100 mg/kg, po) for 21 days protected the rats against 3-nitropropionic acid-induced MCI, which was assessed by novel object recognition and elevated plus maze tests (Sharma et al., 2021). In addition, APP/PS1 double transgenic mouse model was crossbred between PRP-hAPPK595N/M596L single transgenic dementia mouse model and PrP-hPS1dE9 single transgenic dementia mouse model. An increasing number of witnesses have proven that APP/PS1 transgenic mice embody similar pathological changes to those of patients with AD and the memory impairment appears at 4–6 months of age and progresses in an age-dependent manner in APP/PS1 transgenic mice (Herran et al., 2015). Zhong et al. (2018) proved that treatment with EA (50 mg/kg for 60 consecutive days) ameliorated the MCI in APP/PS1 mice assessed by utilizing the Morris water maze test. As a major metabolite of EA, it is demonstrated that urolithin A also ameliorated MCI in APP/PS1 mice based on the Morris water maze test (Gong et al., 2019).

### Behavioral Effects of Ellagic Acid in Models of Scopolamine- and Diazepam-Induced Mild Cognitive Impairment

The clinical features of neurodegenerative diseases such as AD and PD are a gradual loss of cognitive ability, affecting daily activities and learning and memory dysfunction. It is well known that myoglobin acetylcholinergic receptor antagonists impair learning and memory function and this impairment can be replaced by the non-selective antagonist scopolamine. Therefore, scopolamine can be used effectively in amnesic animal models while also inhibiting the activity of central cholinergic neurons (Balmus and Ciobica, 2017; Skalicka-Wozniak et al., 2018). Diazepam can also be used in the construction of this model. Up till now, the remedy for cognitive abnormalities such as attention deficit and amnesia is still far from being identified in medicine, while cognitive-enhancing drugs such as piracetam, memantine, aniracetam, and cholinesterase inhibitors are being applied for ameliorating memory, mood, and behavior; at the same time, the side effects of cognitive impairment have restricted these agents’ usage. The animals administered EA at doses of 30 and 100 mg/kg for 10 days substantially antagonized the MCI induced by scopolamine or diazepam in the elevated plus-maze test in mice and rats, respectively (Mansouri et al., 2016). Also, EA from *Tetrapleura tetraptera* and *Quassia undulata* (50 and 300 mg/kg) ameliorated the MCI induced by scopolamine in rats (Odubanjo et al., 2018).

## Molecular Mechanism of Therapeutic Properties of Ellagic Acid in Mild Cognitive Impairment

**FIGURE 3:**
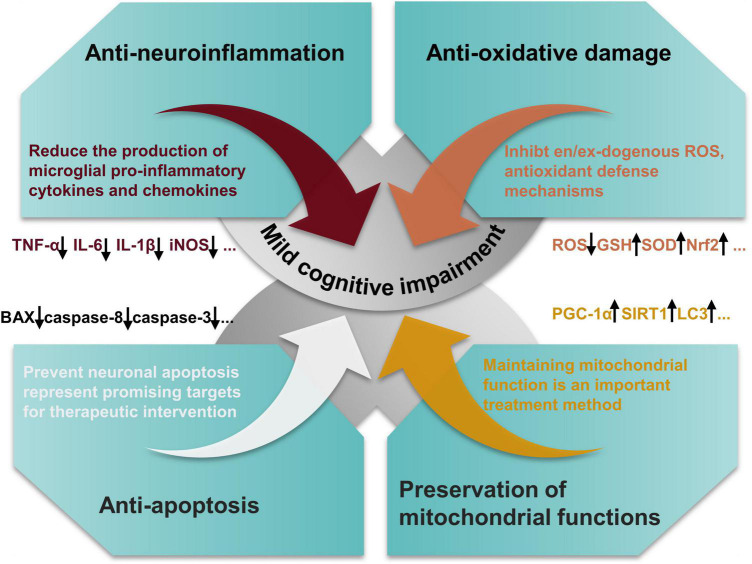
Salient pharmacological features of ellagic acid (EA) elicited by involving anti-oxidant effects, modulation of synthesis of anti-inflammatory molecules, inhibition of neuronal apoptosis, and preservation of mitochondrial functions.

The molecular mechanisms by which EA prevented or reduced MCI vary and include antioxidative damage, modulation of synthesis of antineuroinflammation molecules, inhibition of apoptosis, preservation of mitochondrial functions, and synapse stabilization and plasticity relevant for MCI (Figure 3).

### Antineuroinflammation

Neuroinflammation has been proven to be involved in the pathogenesis of aging and neurodegenerative diseases (Bradburn et al., 2019). Over the decades, a large number of studies on animal models and human beings implies that the immune system plays a fundamental part in learning, memory modulation, and neural plasticity (Yirmiya and Goshen, 2011). Under regular quiescent circumstances, immune mechanisms positively regulate neural circuit remodeling, the promotion of memory functions, and neurogenesis. Nevertheless, when the immune system is vigorously stimulated by infection, harm, or chronic stress state, the inflammatory mediators interrupt the sensitive balance required for neurophysiological actions and result in damaging effects on neural plasticity, neurogenesis, and memory. EA and its microbial metabolites can perform the role of the potential anti-inflammatory agent, mainly by reducing the production of microglial proinflammatory cytokines and chemokines such as tumor necrosis factor-α (TNF-α), interleukin (IL)-6, or interleukin-1β (IL-1β) in the brain tissue in the alleviation of neuroinflammatory responses and prevention of MCI. Alagan et al. (2019) showed that EA from *Phyllanthus amarus* can decrease the release of proteins such as IL-1β, TNF-α, and nitric oxide synthase (iNOS) in LPSs -induced MCI and neuroinflammation. Notably, in rat models of moderate traumatic brain injury, treatment of EA has significantly decreased the proinflammatory cytokine such as TNF-α in brain tissue (Mashhadizadeh et al., 2017). In addition, urolithin A, as the major gut-microbial metabolite of EA, has been revealed to have anti-inflammatory properties *in vitro* and *in vivo*. Gong et al. (2019) indicated that urolithin A not only reduced the levels of activated astrocytes and microglia in the mouse model of AD, but also involved in vital cell signaling pathways, explicitly by enhancing cerebral AMP-activated protein kinase (AMPK) activation, decreasing the activation of P65NF-κB and P38MAPK.

### Antioxidative Damage

Free radical-mediated oxidative impairment of biological molecules has been connected with the pathogenesis of several neurodegenerative disorders (Kahroba et al., 2021). Particularly, a surfeit of lipid peroxidation and depletion of brain antioxidant defense ruin neuronal coherence and cognitive prowess. Antioxidants are proposed to modify or decelerate the progression of neurodegenerative disease pathogenesis and clinical symptoms such as MCI (Chauhan and Chauhan, 2020). EA possesses excellent antioxidant activities, as the two pairs of the hydroxyl group exist in the structure to enhance its antioxidant properties. In fact, EA comprises two lactone and four functional phenolic groups linked to involvement in redox reactions and thereby it can protect crucial molecules from hazardous free radicals (Alfei et al., 2020). Meanwhile, the gene expression of molecules that are engaged in antioxidant defense can be activated by EA (Zhang et al., 2022). In the same way, the expression of proteins, enzymes, and transcription factors that are participating in oxidative stress-producing pathways can be repressed by EA such as cytochrome P450-dependent phase-I enzymes and nicotinamide adenine dinucleotide phosphate oxidase. Previous findings confirmed that administration of EA prevents cognitive impairment through attenuation of the brain lipid peroxidation and prevented waning of the superoxide dismutase (SOD), glutathione (GSH), and catalase (CAT) activity in streptozotocin-induced rats (Kumar and Bansal, 2018). Cao et al. (2020) revealed that ellagitannin activates AMP-activated protein kinase (AMPK) to ameliorate oxidative stress by activation of NF-E2-related factor (Nrf2)-mediated phase II antioxidant enzymes. In such a manner, EA is involved not only in the management of endogenous and exogenous sources of reactive oxygen species (ROS), but also involved in antioxidant defense mechanisms.

### Antiapoptosis

The internal signals of the mitochondria can exactly mediate apoptosis, a programmed cell death mechanism, or the extrinsic signals received to the cell surface (death domain triggering) receptors (Bedoui et al., 2020). Neuronal apoptosis is a vital attribute of neurodegenerative disease pathology. Lots of evidence indicated that preventing apoptosis also represents a promising target for therapeutic intervention. As an example, some recent studies suggested that markers for apoptotic cell death-like caspases are activated in striatal neurons from transgenic animals and patients with HD (Chongtham et al., 2021). An *in-vivo*-based study reported that treatment of EA of R6/2 mice significantly reduced the apoptotic neurons by downregulating Bax and caspase-8 in the striatum and cortex (Sun et al., 2020). Notably, an *in-vivo* study of APP/PS1 transgenic mice and its amelioration by EA corroborated results on cleaved caspase-3 activation (Zhong et al., 2018). Interestingly, in a similar study, EA from immature persimmon (*Diospyros kaki*) ameliorated the cognitive dysfunction and downregulated the expression of Bax and caspase-3 in (Aβ)_1–42_-induced Institute for Cancer Research (ICR) mice (Yoo et al., 2021). EA can prevent MCI by regulating Phosphoinositide 3 (PI3)-kinase-endothelial nitric oxide synthase (eNOS) signaling and thereby regulating cellular apoptosis in streptozotocin-treated rats (Kumar and Bansal, 2018). Meanwhile, urolithin A, the metabolite of EA, attenuated the D-galactose-induced neuronal apoptosis in aging mice by regulating the sirtuin 1/mammalian target of rapamycin (SIRT1/mTOR) signaling pathway (Chen et al., 2019).

### Preservation of Mitochondrial Functions

Mitochondria generate the basic ATP for the optimal survival function of neurons and the accumulation of damaged mitochondria is a distinguishing feature of aging and age-related neurodegenerative disorders (Grimm and Eckert, 2017). Especially, mitochondrial dysfunction is a profound phenomenon in AD as it occurs in human samples of both the familial and sporadic types of the disease, as well as in transgenic AD mouse models’ brain tissues (Fang et al., 2019). Mitochondrial quality control is regulated according to the procedures of mitophagy and mitochondrial biogenesis (Annesley and Fisher, 2019). Multiple evidence suggested that EA and its derivatives such as urolithin A restore defective mitochondria in a significant manner for MCI therapy by modulating mitochondrial biogenesis and rescuing dysfunctional autophagy (de la Torre et al., 2020; Jayatunga et al., 2021). A recent study showed that urolithin A inhibits high glucose-induced neuronal amyloidogenesis by regulating transglutaminase type 2-dependent endoplasmic reticulum-mitochondria contacts and calcium homeostasis (Lee et al., 2021). Meanwhile, urolithin A activates mitochondrial autophagy to exert neuroprotective effects through activation of the SIRT1/mTOR signaling pathway, which aids in preventing D-galactose-induced brain aging (Chen et al., 2019). Cao et al. (2020) found that punicalagin regulates mitochondrial biogenesis by activating AMPK/peroxisome proliferator-activated receptor gamma coactivator 1-α (PGC-1α) signaling, thereby preventing cognitive dysfunction. Additionally, mitochondrial respiratory dysfunction is also the main cause of ROS overproduction and treatment of EA can prevent the decrease of mitochondrial succinate dehydrogenase activity in the rat brain caused by 3-nitropropionic acid, thus protecting mitochondrial functions (Sharma et al., 2021).

## Conclusion and Future Prospects

**FIGURE 4:**
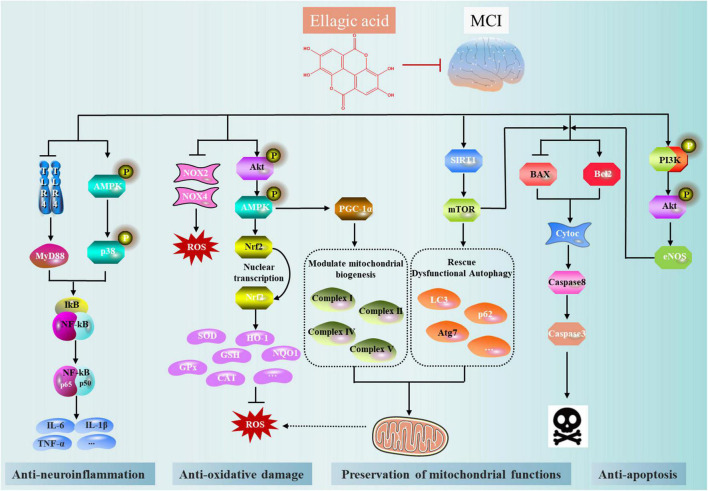
The main mechanism network of anti-mild cognitive impairment (MCI) effect of ellagic acid (EA).

Multiple evidences indicate that plant-derived EA and its derivatives existing in natural sources such as kakadu plum, pomegranate, and raspberry are natural bioactive compounds with considerable favorable health properties. Especially, EA has been accepted to be an existing food additive in Japan and is principally regarded as an antioxidant that can be utilized without any toxicological data or any limitations in respect of the concentration utilization limit. So far, the preclinical studies of EA and its derivatives conducted on various cell lines and animal models facilitated the idea of dietary EA as a feasible agent to be able to the specifical target and improve MCI. In addition, we found a wide range of doses of EA in these behavioral experiments, ranging from a minimum of 25 mg/kg to a maximum of 300 mg/kg. We speculated that this might be related to the strains of animals selected in the experiments and their physical conditions. But happily, all the witnesses mentioned above suggest that EA has little toxicity to tissue or normal cells. EA and its derivatives exert beneficial effects on MCI by reducing the ability of neuroinflammation, oxidative stress, neuronal apoptosis, synaptic dysfunction and loss, and defective mitochondrial functions (Figure 4). A randomized double-blind clinical trial found that EA could improve sleep quality and gastrointestinal symptoms in patients with irritable bowel syndrome and its antioxidant and anti-inflammatory properties may be the main reason for this effect (Mirzaie et al., 2021). In addition, another clinical trial study confirmed that supplements of EA can be used as dietary supplements in patients with type 2 diabetes by improving chronic adverse effects (Ghadimi et al., 2021). It has also been shown to reduce metabolic disorders in women (Kazemi et al., 2021). In conclusion, EA, as a dietary supplement, plays an important role in the treatment of many diseases. Nevertheless, there are no large randomized controlled clinical trials of EA for the treatment of MCI. Although a large amount of preclinical data has been accumulated on the use of EA for the treatment of MCI, the clinical research evidence, particularly the large randomized controlled trials, on the topic is still scarce. This emphasizes the well-designed and correctly powered randomized controlled trials in the human population that need to be performed to reinforce the scientific facticity of the beneficial effects of EA on MCI. Synchronously, the mechanism of EA against MCI is least provided cynosure and expects more attention from the emerging research community. In particular, the emergence of new technologies and methods, such as genomics, metabolomics, proteomics, and bioinformatics network analysis techniques (Hou et al., 2021; Wang et al., 2022), has provided support for the screening of potential targets and revealing the mechanism of action of EA in the treatment of MCI. All in all, drug development from these innate resources ought to be encouraged and upgraded, keeping in mind its effectiveness and capability to perform the role of a safe drug, thereby improving the quality of human life and promoting public health to manage MCI.

## Author Contributions

YZ, XM, and WW conceived and proposed the idea. WW designed the research and wrote the manuscript. SW revised and edited the illustrations and words in the manuscript. XW and YL revised the manuscript. All authors have read the final version of the manuscript.

## Conflict of Interest

The authors declare that the research was conducted in the absence of any commercial or financial relationships that could be construed as a potential conflict of interest.

## Publisher’s Note

All claims expressed in this article are solely those of the authors and do not necessarily represent those of their affiliated organizations, or those of the publisher, the editors and the reviewers. Any product that may be evaluated in this article, or claim that may be made by its manufacturer, is not guaranteed or endorsed by the publisher.
